# Synthesis and Protective Effect of Scutellarein on Focal Cerebral Ischemia/Reperfusion in Rats

**DOI:** 10.3390/molecules170910667

**Published:** 2012-09-06

**Authors:** Lihua Qian, Minzhe Shen, Hao Tang, Yuping Tang, Li Zhang, Yifan Fu, Qianping Shi, Nian-Guang Li

**Affiliations:** 1 Jiangsu Key Laboratory for High Technology Research of TCM Formulae, Nanjing University of Chinese Medicine, Nanjing 210046, China; 2 Department of Medicinal Chemistry, Nanjing University of Chinese Medicine, Nanjing 210046, China

**Keywords:** scutellarein, scutellarin, synthesis, MCAO, focal cerebral ischemia/reperfusion

## Abstract

Scutellarein, the main metabolite of scutellarin *in vivo*, has relatively better solubility, bioavailability and bio-activity than scutellarin. However, compared with scutellarin, it is very difficult to obtain scutellarein from Nature. Therefore, the present study focused on establishing an efficient route for the synthesis of scutellarein by hydrolyzing scutellarin. Neurological deficit score and cerebral infarction volume with the administration of scutellarein were then used to compare its neuroprotective effects on focal cerebral ischemia/reperfusion in rats induced by middle cerebral artery occlusion (MCAO) with those of scutellarin. The results showed that scutellarein had better protective effect on focal cerebral ischemia/reperfusion than scutellarin, which laid the foundation for further research and development of scutellarein as a promising candidate for ischemic cerebro-vascular disease.

## 1. Introduction

Cerebrovascular disease is a common and frequently-occurring disease that seriously endangers human health. It is one of the leading causes of death and disability worldwide, especially ischemic cerebrovascular disease, which is the most frequently prevalent [1]. Scutellarin (4°,5,6-trihydroxyflavone-7-glucuronide), the major active component in breviscapine extracted from the Chinese herb *Erigeron breviscapus* (vant.) Hand.-Mazz., has been clinically used in China since 1984 to treat acute cerebral infarction and paralysis induced by cerebrovascular diseases such as hypertension, cerebral thrombosis, cerebral haemorrhage [2]. Although it has been clinically used for a long time, scutellarin has low solubility [3,4], poor oral absorption and low bioavailability [5]. Some researchers found that scutellarin was mainly absorbed in the form of its hydrolyzed product scutellarein by the intestine [6], and scutellarein was much more easily absorbed, with triple the bioavailability, after oral administration of scutellarin and scutellarein in equal amounts [7]. Furthermore, in the clinical trials [8], a large amount of scutellarein was found in urine and plasma after oral administration of breviscapine in subjects, indicating that breviscapine was firstly hydrolyzed into the aglycone when reaching the colon and was then absorbed in the form of scutellarein as the real bioactive component in the body. Pharmacodynamics confirmed that scutellarein had strong *in vitro* antioxidant activity [9] and scutellarein pretreatment could ameliorate rat brain injury after ischemia*in vivo*, with better activity than scutellarin [10].

There is little scutellarein in *E. breviscapus* compared with large amounts of scutellarin [10]. Frakas [11] and Cui [12] had already completed the total synthesis of scutellarein, but the route was long and the yield was low. Thus, we tried the synthesis of scutellarein by hydrolyzing scutellarin in water. Unfortunately, it was found that they did not react. As a result, this study was intended to establish an efficient route to the synthesis of scutellarein by hydrolyzing scutellarin, and to study its protective effect on focal cerebral ischemia/reperfusion in rats in comparison with scutellarin, which will guide the search for more potent protective agents for ischemic cerebrovascular disease.

## 2. Results and Discussion

### 2.1. Optimization of Reaction Conditions for the Synthesis of Scutellarein

The optimization of reaction conditions is shown in Table 1. Concentrated sulfuric acid was selected as a catalyst. Firstly, 0.5 g of scutellarin was added to 10 mL of 1 mol/L H_2_SO_4_ in water, and the reaction was performed at 90 °C for 6~24 h under a N_2_ atmosphere. Due to the poor water solubility of scutellarein no product was formed under these conditions, even the concentration of H_2_SO_4_ was increased from 1 mol/L (Table 1, run 1) to 3 mol/L (Table 1, run 3). Then, the water solvent was changed for ethanol, where scutellarein had good solubility. After several attempts, we found that increasing the concentration of ethanol (Table 1, run 4–6) accelerated its hydrolysis and the yield increased to 2.1% in 90% ethanol (Table 1, run 6). Subsequently, the concentration of H_2_SO_4_ was optimized. When the concentration of H_2_SO_4_ was increased from 1.0 mol/L (Table 1, run 7) to 3.0 mol/L (Table 1, run 9), the yield of scutellarein was improved from 5.3% to 10.0%, and the yield could be further increased to 12.1% when the reaction time was extended to 48 h (Table 1, run 10). Lastly, the exogenous reaction temperature was studied in this reaction. When the reaction temperature was raised from 90 °C (Table 1, Run 10) to 120 °C (Table 1, Run 12), the yield of scutellarein increased from 12.1% to 17.3%. As a result, 3.0 mol/L H_2_SO_4_ in 90% ethanol and heating under a N_2_ atmosphere at 120 °C for 48 h were selected as the best conditions for the synthesis of scutellarein.

**Table 1:** Optimization of reaction conditions in the synthesis of scutellarein by hydrolyzing scutellarin.

Run	Reaction conditions	Yield (%)
**1**	1.0 mol/L H_2_SO_4_ in water, 90 °C, 6~24 h	No product
**2**	2.0 mol/L H_2_SO_4_ in water, 90 °C, 6~24 h	No product
**3**	3.0 mol/L H_2_SO_4_ in water, 90 °C, 6~24 h	No product
**4**	0.5 mol/L H_2_SO_4_ in 70% ethanol, 90 °C, 6~24 h	No product
**5**	0.5 mol/L H_2_SO_4_ in 80% ethanol, 90 °C, 6~24 h	No product
**6**	0.5 mol/L H_2_SO_4_ in 90% ethanol, 90 °C, 24 h	2.1
**7**	1.0 mol/L H_2_SO_4_ in 90% ethanol, 90 °C, 24 h	5.3
**8**	2.0 mol/L H_2_SO_4_ in 90% ethanol, 90 °C, 24 h	8.5
**9**	3.0 mol/L H_2_SO_4_ in 90% ethanol, 90 °C, 24 h	10.0
**10**	3.0 mol/L H_2_SO_4_ in 90% ethanol, 90 °C, 48 h	12.1
**11**	3.0 mol/L H_2_SO_4_ in 90% ethanol, 100 °C, 48 h	15.2
**12**	3.0 mol/L H_2_SO_4_ in 90% ethanol, 120 °C, 48 h	17.3

### 2.2. Neurological Deficit Score

As shown in Table 2, no neurological symptoms were observed in any rats with sham operation. The middle cerebral artery occlusion (MCAO) group obviously had neurological deficit (*p* < 0.01); the rats could not act as spontaneously as the normal group. However, the rats pretreated with scutellarein showed a dose-dependent decreased neurological deficit score, and the effect of scutellarein (100 mg/kg) was better than that of scutellarin (100 mg/kg, *p* < 0.05).

**Table 2:** The effect of scutellarein on neurological scores and cerebral infarct volume in MCAO rats (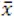 ± *s*, *n* = 10).

Group	Dose (mg/kg)	Behavioral Scores	Cerebral Infarction Volume (%)
Sham	−	0.00 ± 0.00	0.00 ± 0.00
MCAO	−	3.00 ± 0.53 ^##^	22.24 ± 3.91 ^##^
Scutellarin	100	2.16 ± 0.41 **	15.81 ± 1.48 **
Scutellarein	100	1.33 ± 0.69 **^◆^	9.66 ± 4.29 **^◆^
	50	2.25 ± 0.46 **	15.91 ± 6.04 *
	25	2.50 ± 0.55	19.04 ± 7.21

** *p* < 0.01, * *p* < 0.05 *vs*. MCAO group; ^##^*p* < 0.01 *vs*. Sham operation group; ^◆^ *p* < 0.05 *vs.* Scutellarin group.

### 2.3. Cerebral Infarction Volume

**Figure 1:**
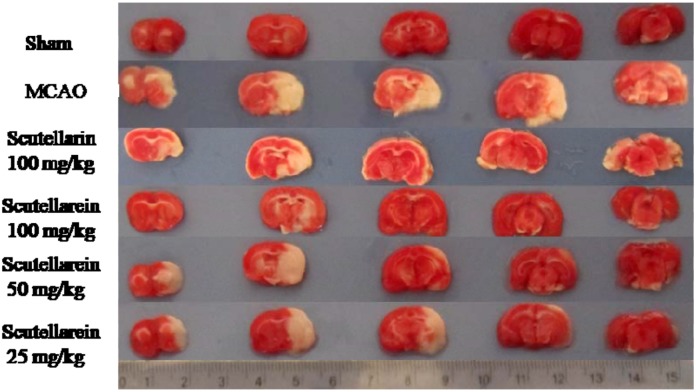
The effect of scutellarein on cerebral infarction volume by TTC staining.

None of the rats in the sham operation group exhibited infarction volume. In contrast, there was a significant difference between the sham and the MCAO groups (*p* < 0.01), as shown in Table 2 and Figure 1. Most area of the right hemisphere could not be stained red in the MCAO group rats. However, the rats pretreated with scutellarein could dose-dependently decrease the infarction volume, and the effect of scutellarein (100 mg/kg) was better than scutellarin (100 mg/kg, *p* < 0.05). The protective effect of scutellarein might be due to its cerebral neuroprotection by reducing cell apoptosis [13].

MCA is *pars affecta* easily caused by ischemic cerebrovascular disease in the clinic. MCAO takes up a large proportion of the clinical first cerebral apoplexy, leading to the symptoms of contralateral hemiplegia, hemianesthesia, and so on [14]. The MCAO rat model was the animal model which is most commonly used in library studies because it’s pathological process was very similar to that of clinical cerebral apoplexy. Neurological deficit score and cerebral infarct volume are dependable indexes for evaluation of cerebral ischemic injury, therefore, they are commonly used to study the therapeutical effect of drugs on cerebral ischemic injuries. The pharmacological results of the present study showed that scutellarein had better protective effects on cerebral ischemic injury than scutellarin on the same dose, which was also consistent with the literature [10]. It was reported that scutellarin had low solubility [3,4], poor oral absorption and low bioavailability [5], and the absolute bioavailability of scutellarein was 7.0% after oral administration, while the bioavailability of scutellarein was 301.8%, because scutellarein could be absorbed more easily than scutellarin [7]. Additionally, the reported pharmacokinetic behavior results of scutellarin showed that scutellarin might be hydrolyzed by intestinal β-glucuronidase of bacterial origin, followed by a reconjugation step in the intestinal cell and/or in the liver with glucuronic acid after absorption of the aglycone, which showed that scutellarin had positional selectivity [15]. In humans, the 6-OH group of the aglycone of scutellarin was the preferential site for glucuronosyl conjugation compared with 4-, 5-, and 7-OH groups [15]. All of these results indicated that scutellarein, the main metabolite of scutellarin *in vivo*, has relatively better solubility, bioavailability and bio-activity than scutellarin.

## 3. Experimental

### 3.1. Materials and Subjects

Scutellarin was purchased from Mianning Jiexiang Co. Ltd. (Chengdu, China). 2,3,5-Triphenyltetrazolium chloride (TTC) was purchased from Sigma Chemical Co. (Shanghai, China). ^1^H-NMR spectra were obtained using a Bruker AV-300 (300 MHz, Billerica, MA, USA) and AV-500 (500 MHz). ESI-MS spectra were recorded on a Waters Synapt HDMS spectrometer (Manchester, UK). Sixty male Spargue-Dawley rats (240–280 g) obtained from Shanghai Slac Laboratory Animal Co. Ltd. (Shanghai, China) were used. Animals were bred in a breeding room with temperature of 24 ± 2 °C, humidity of 60 ± 5%, and 12 h dark-light cycle. They were given tap water and fed normal food *ad libitum*. Animal welfare and experimental procedures were strictly in accordance with the *Guide for the Care and Use of Laboratory Animals* (US National Research Council, 1996) and the related ethics regulations of our University.

### 3.2. General Procedure for the Synthesis of Scutellarein

All the reagents were commercially available and used directly. Air- and moisture-sensitive liquids and solutions were transferred via syringe or stainless steel cannula. Organic solutions were concentrated by rotary evaporation below 45 °C at approximately 20 mm Hg. All non-aqueous reactions were carried out under anhydrous conditions using flame-dried glassware within an argon atmosphere in dry and freshly distilled solvents, unless otherwise noted. Reactions were monitored by thin-layer chromatography (TLC) carried out on 0.15~0.20 mm Yantai silica gel plates (RSGF 254) using UV light as the visualizing agent. Chromatography was performed on Qingdao silica gel (160~200 mesh) using petroleum ether (60~90) and ethyl acetate as the eluting solvent.

**Scheme 1:**
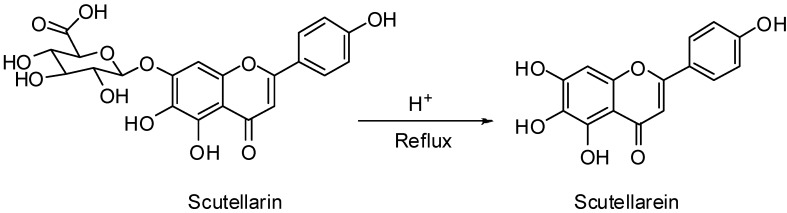
Synthesis of scutellarein.

Scutellarin (0.5 g) was added to solutions of H_2_SO_4_ in 0~90% ethanol (10 mL, 0.5~3 mol/L). Then the reaction mixture was refluxed at 90~120 °C (outside temperature) in N_2_ atmosphere for 6~48 h. After cooling to 25 °C, the reaction mixture was added to ice water; the solid was filtered and then recrystallized with 90% ethanol to give the target compound scutellarein. ^1^H-NMR (300 MHz, DMSO-*d*_6_) *δ*: 6.78 (1H, s), 6.73 (1H, s), 6.90–6.93 (2H, d, *J* = 8.8 Hz), 7.90–7.93 (2H, d, *J* = 8.8 Hz), 8.71 (1H, s), 10.30 (1H, s), 10.44 (1H, s), 12.79 (1H, s). ESI-MS (negative): *m/z* 285 [M−H]^−^. The synthesis route of scutellarein was outlined in Scheme 1. Detailed synthesis conditions are listed in Table 1.

### 3.3. Animal Model of MCAO

The rats were randomly divided into six groups (n = 10/group): sham operation group; MCAO group without pretreatment; MCAO group pretreated with scutellarin (100 mg/kg); MCAO groups pretreated with different dose of scutellarein (100, 50, 25 mg/kg [10]). Scutellarein and scutellarin were dissolved in 0.9% saline water. After intragastric administration for six days, the rats were anesthetized with 10% chloral hydrate (i.p. 300 mg/kg) on the seventh day. The MCAO rat model was established by inserting a fishing line coated with silicon (diameter 0.32 mm) from the right external-internal carotid artery (ECA-ICA) into the middle cerebral artery (MCA) for 2 h occlusion followed by 24 h reperfusion according to the method in Longa *et al*. [16,17].

### 3.4. Neurological Deficit Score

Neurological deficit was assessed 24 h after reperfusion according to a modified method established by Bederson [18] as follows: 0, no neurological symptoms; 1, unable to completely extend the front jaw on the contralateral side; 2, rotating and crawling to the contralateral side; 3, dumping to the contralateral side; 4, unable to walk spontaneously.

### 3.5. Measurement of Cerebral Infarction Volume

The cerebral infarct volume was measured using TTC staining [19]. All the rats were decapitated after neurological deficit scoring. Their brains were quickly removed, frozen to a solid at −20 °C, and then cut into 2-mm-thick coronal slices. Five selected sections were stained in a 2% solution of TTC at 37 °C for 30 min. Normal cerebral areas could be stained to be red, but the infarction areas could not be stained and showed pale. The infarction volume was calculated as infarction rate (%) = *A_0_/A'* × 100%, where *A_0_* was the weight of the infarction areas, was the weight of the whole brain [20,21].

## 4. Conclusions

In summary, an efficient route for the synthesis of scutellarein by hydrolyzing scutellarin was reported. Essential to the synthesis was the use of H_2_SO_4_ in 90% ethanol under a N_2_ atmosphere. The synthesis method solved the problem of the low yield of scutellarein, and provided a possible route for its industrialized production. The MCAO assay clearly demonstrated that scutellarein had better neuroprotection effects than scutellarin on focal cerebral occlusion/reperfusion by decreasing neurological deficit score and cerebral infarction volume, which suggested that scutellarein would be a more promising potent agent for the therapy of ischemic cerebrovascular disease.
